# Peritumoral Edema Is Associated With Postoperative Hemorrhage and Reoperation Following Vestibular Schwannoma Surgery

**DOI:** 10.3389/fonc.2021.633350

**Published:** 2021-03-09

**Authors:** Xiaoming Guo, Yueli Zhu, Xiaoyu Wang, Ke Xu, Yuan Hong

**Affiliations:** ^1^Department of Neurosurgery, The Second Affiliated Hospital, Zhejiang University School of Medicine, Hangzhou, China; ^2^Department of Neurosurgery, Tongde Hospital of Zhejiang Province, Hangzhou, China; ^3^Department of Geriatrics, The First Affiliated Hospital, Zhejiang University School of Medicine, Hangzhou, China

**Keywords:** reoperation, hemorrhage, cerebellopontine angle, adverse event, peritumoral edema, vestibular schwannoma

## Abstract

**Background:** Postoperative hemorrhage (POH) is a severe complication following vestibular schwannoma surgery that may require surgical treatment. The purpose of our study is to identify risk factors associated with POH and reoperation following the resection of vestibular schwannoma.

**Methods:** We retrospectively recruited 452 vestibular schwannoma patients treated with retrosigmoid approach. The primary outcome was POH, and the secondary outcome was reoperation for POH. Clinical and radiographic data were compared by performing univariate analysis and logistic regression analysis.

**Results:** Among the 452 patients, 37 patients (8.2%) presented with POH and14 patients (3.1%) required reoperation within a 30-day hospitalization period. The univariate analysis showed that peritumoral edema, tumor diameter >30 mm, severe postoperative hypertension, and length of hospital stay were associated with POH and reoperation for POH. Logistic regression analysis showed that peritumoral edema [odds ratio (OR) 4.042, 95% confident interval (CI) 1.830–8.926, *P* = 0.001] and tumor diameter >30 mm (OR 3.192, 95% CI 1.421–7.168, *P* = 0.005) were independent predictive factors for POH. Peritumoral edema (OR 7.071, 95% CI 2.342–21.356, *P* = 0.001) was an independent predictive factor for reoperation by using logistic regression analysis. Further analysis revealed that larger tumor and incomplete tumor resection were both associated with a higher incidence of peritumoral edema.

**Conclusion:** Peritumoral edema and tumor size are independent risk factors for POH following vestibular schwannoma surgery. And larger hematoma occurs more commonly in tumors with peritumoral edema which may require reoperation. Tumor size and extent of tumor resection are associated with peritumoral edema. Close attention should be paid to high-risk patients especially for those who presented with severe postoperative hypertension.

## Introduction

Vestibular schwannoma (VS) is a common benign tumor of posterior cranial fossa. With the evolution of VS resection technology, the procedures are most often safe and well-tolerated (1, 2). Recent decades, a large volume of studies focused on facial, acoustic and other nerve function preservation after the resection of VS while few articles were specifically dedicated to describing postoperative hemorrhage (POH). However, this complication was not uncommon with an incidence varying from 1.7 to 8% (2–5). POH can mainly cause obstruction of the fourth ventricle which would result in intracranial hypertension. Therefore, postoperative intracranial hemorrhage was one of the major causes of emergency reoperation and significant morbidity (1, 3, 6, 7). However, to date, the studies analyzing the risk factors for POH and reoperation are still limited. There have been two studies that explored the relationship between perioperative factors and reoperation (8, 9). However, radiological features of VS were not included in both two studies.

The purpose of our study is to identify clinical characteristics and radiological features which are associated with POH and unplanned reoperation following the resection of vestibular schwannoma. It is of great value to pay more attention to the risk factors for neurosurgeons to reduce the incidence of POH and reoperation which can further improve surgical safety.

## Materials and Methods

### Patients

**Figure 1:**
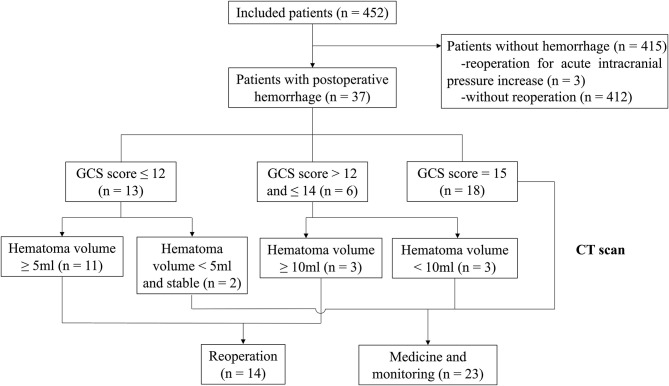
Flow diagram depicting the study.

This study enrolled 452 patients with vestibular schwannoma from the Second Affiliated Hospital of Zhejiang University School of Medicine between January 2016 and November 2019. Patients over 18 years of age who were diagnosed as vestibular schwannoma by magnetic resonance imaging (MRI) and pathology, were included. All patients should be treated via retrosigmoid approach in the lateral position. Patients treated by middle fossa approach and translabyrinthine approach were excluded. Patients without preoperative radiological data were also excluded. Patients were grouped according to the presence or absence of POH, which served as the hemorrhage group and non-hemorrhage group, respectively. Furthermore, patients that required reoperation due to POH were categorized into the reoperation group, and rest of the patients were categorized into the non-reoperation group. The decision of reoperation was made based on the deterioration of vital signs, worsening of neurological symptoms, and some computed tomography (CT) findings, including hematoma volume, compression of basal cistern and acute hydrophalus. The flow diagram was shown in Figure 1. All patients had CT images on postoperative day 1, as well as MRI on postoperative day 3, which were used to identify whether there was bleeding and to quantify the extent of tumor resection. Only intermittent pneumatic compression was applied for the prevention of thrombo-embolic complications. Except in 3 patients, they had been treated with antiplatelet and anticoagulant drugs due to the history of cerebral infarction or atrial fibrillation. Antiplatelet and anticoagulant treatments were discontinued about 4–7 days before procedures, and were resumed 1–2 weeks after procedures. This study was approved by the Human Ethics Review Committee of the Second Affiliated Hospital, Zhejiang University School of Medicine.

### Variables and Definitions

All patients who met the criteria were included in our study. Demographic parameters [age, sex, body mass index (BMI)], length of hospital stay, tumoral characteristics [maximum tumor diameter, cystic vestibular schwannoma, peritumoral edema (PTE), and extent of tumor resection], past medical history (hypertension, diabetes mellitus, smoking history, drinking history, antiplatelet use, and anticoagulant use), and postoperative clinic data [international normalized ratio (INR), central nervous system infection (CNS), unplanned reoperation, blood pressure, and in-hospital death] were recorded for each case. For the hemorrhage group and reoperation group, hematoma volume, hematoma location, surgical approach, postoperative Glasgow Coma Scale scores, and clinical signs, and symptoms revealing hematoma were also recorded.

**Figure 2:**
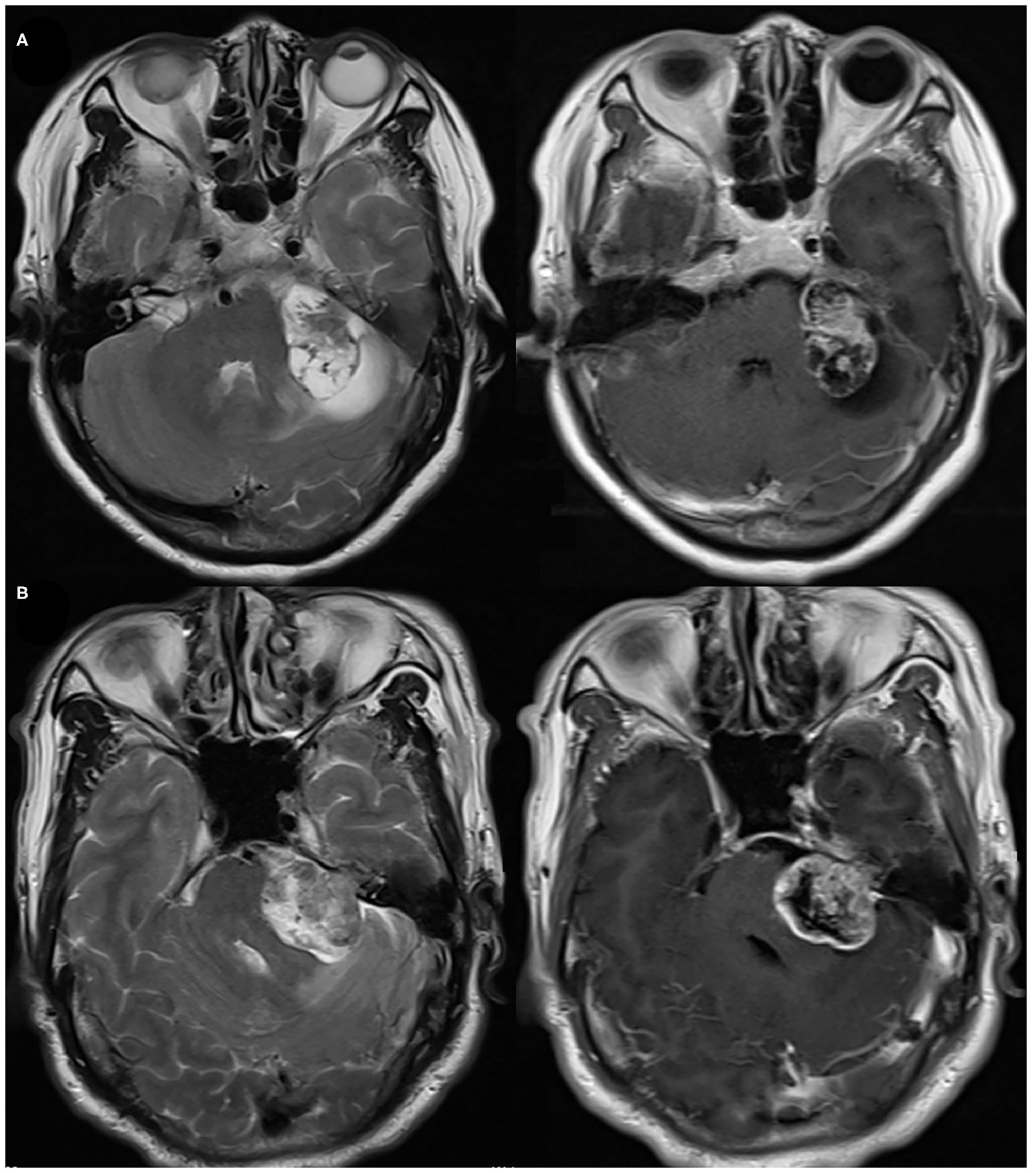
**(A)** Preoperative axial T2 and enhanced T1 images of brachium pontis and cerebellar edema. **(B)** Preoperative axial T2 and enhanced T1 images of cerebellar edema.

The tumor size was measured at the maximum tumor diameter of the CPA portion by preoperative enhanced MRI in the axial slice across the internal auditory canal (10). A size of >30 mm was defined as large VS (7). PTE was identified on fluid attenuated inversion recovery (FLAIR) and T2-weighted sequence at following sites: cerebellum, brainstem, and brachium pontis (Figure 2). Cystic vestibular schwannoma was identified by FLAIR and enhanced sequence. Postoperative MRI and CT were reviewed in all patients for identifying the presence of POH. Hematoma volume was estimated by surgical record or Coniglobus formula (ABC/2) on postoperative CT or postoperative MRI. Hematoma volume ≥1 mL was defined as POH. Severe postoperative hypertension was defined as the presence of systolic blood pressure above 180 mmHg and using intravenous antihypertensive drugs. The CNS infection was diagnosed according to Chinese expert consensus on diagnosis and treatment of critically infection in neurosurgery (2017). All radiological data was recorded by two independent neurosurgeons (X.G. and X.W.). All disagreements were resolved by a senior neurosurgeon (Y.H.).

### Statistical Analysis

All statistical analyses were performed with SPSS version 22.0 (SPSS for Mac, IBM Corp). For continuous variables, differences between groups were performed using Student's *t*-test or Mann–Whitney *U*-test. Categorical data were analyzed with a chi-square test or Fisher's exact test. Variables with significance of *P* < 0.1 in the univariate analyses were then included in the binary logistic regression analyses to determine independent factors that were associated with an increased risk of POH and unplanned reoperation. For all tests, a *P*-value of < 0.05 was considered statistically significant.

## Results

### Patient and Treatment Demographics

A total of 452 patients were enrolled in this study between January 2016 and November 2019. The median age (± interquartile range, IQR) was 53.0 ± 16.0 years (range 24–82 years), and the median BMI (± IQR) was 23.0 ± 4.2 kg/m^2^ (range 16.0–35.6 kg/m^2^). The median length of hospital stay (± IQR) was 16.0 ± 7.0 days (range 4–531 days). There was a total of 37 patients with POH which were divided into the hemorrhage group, including 14 of them in the reoperation group and the other 23 patients in the non-reoperation group.

### The Clinical Course of POH and Reoperation

In this study, 37 patients (8.2%) presented with POH and 14 patients (3.1%) underwent an unplanned operation for hematoma. The median hematoma volume (± IQR) in the reoperation group was 15.0 ± 11.0 ml, while it was 2.4 ± 3.7 ml in the rest 23 patients (*P* < 0.001). All patients in the reoperation group presented with decreased level of consciousness. Five patients presented with decreased level of consciousness but without reoperation.

The average time between the first operation and the reoperation was 1.9 days (range 0.1–5.8 days, Table 1). For hematoma locations in the reoperation group, 10 of them were in cerebellopontine angle (CPA); two of them were on the brainstem surface; and two of them were in cerebellum. For the treatment, three patients only underwent hematoma evacuation; five patients underwent hematoma evacuation and decompressive craniotomy; six patients underwent hematoma evacuation, decompressive craniotomy and external ventricular drainage. One patient fell into a persistent coma after the reoperation and one patient died 2 days after the reoperation. Five patients developed hydrophalus and underwent ventriculoperitoneal shunts. CNS infection occurred in three patients (21.4%). The median length of hospital stay (± IQR) in the reoperation group was 27.5 ± 15.3 days (range 10–531 days), which was much longer than the non-reoperation group (16.0 ± 6.0 days, range 4–55 days).

**Table 1:** Clinical courses of the reoperation patients for hematoma.

**No**	**Age (years), sex**	**History of hypertension**	**Tumor size (mm)**	**PTE**	**Extent of resection**	**Hematoma location**	**Hematoma volume (mL)**	**Time interval (days)**	**Postop. GCS scores**	**Clinical signs and symptoms revealing hematoma**	**Treatment**	**Complications**
1	49, F	N	35	Y	STR	Brainstem	15	0.6	8	Bilateral mydriasis	HE+EVD+DC	NA
2	55, M	N	27	N	GTR	CPA	10	2.1	14	Headache, severe hypertension*	HE+DC	NA
3	52, M	N	33	N	STR	CPA	15	5.8	11	NA	HE+DC	Hydrocephalus, reoperation for three times, re-admission, CNS infection
4	62, M	N	44	Y	STR	CPA	15	4.1	14	Hemiparesis, severe hypertension*	HE	Hemiparesis, CNS infection
5	54, M	N	45	Y	GTR	CPA	24	1.8	14	Severe hypertension^#^	HE+EVD+DC	Hydrocephalus, CNS infection
6	38, F	N	42	N	STR	Brainstem	28	0.1	10	SpO^2^ decreased, severe hypertension*	HE+DC	NA
7	62, F	Y	25	N	STR	Cerebellum	20	0.2	4	SpO^2^ decreased	HE+EVD+DC	Persistent coma, Hydrocephalus
8	49, F	N	43	N	GTR	CPA	25	0.2	5	Bilateral mydriasis	HE+DC	NA
9	53, F	Y	36	N	GTR	CPA	15	1.1	6	NA	HE+EVD+DC	Hydrocephalus
10	63, M	N	21	N	GTR	CPA	15	2.0	3	Bilateral mydriasis, severe hypertension*	HE+EVD+DC	Death
11	62, F	N	39	Y	STR	CPA	15	1.9	12	NA	HE	NA
12	75, M	N	41	N	GTR	CPA	20	2.1	12	Severe hypertension*	HE+DC	NA
13	63, M	N	40	Y	GTR	CPA	10	0.2	12	NA	HE	NA
14	62, M	Y	33	Y	STR	Cerebellum	7	3.7	3	Bilateral mydriasis, severe hypertension^#^	HE+EVD+DC	Hydrocephalus

*PTE, peritumoral edema; STR, subtotal resection; GTR, gross total resection; NA, not applied; CPA, cerebellopontine angle; Postop., postoperative; GCS, Glasgow Coma Scale; HE, hematoma evacuation; EVD, external ventricular drainage; DC, decompressive craniectomy*.

* *Five of them had suffered severe postoperative hypertension prior to or simultaneously with deterioration of vital signs or worsening of neurological symptoms*.

# *Two of them had suffered severe postoperative hypertension after aforementioned signs and symptoms*.

### Univariate and Logistic Regression Analysis of POH and Reoperation

As shown in Table 2, the univariate analysis showed that PTE (*P* < 0.001), max tumor diameter (*P* < 0.001), tumor diameter >30 mm (*P* < 0.001), severe postoperative hypertension (*P* < 0.001), and length of hospital stay (*P* < 0.001) were associated with POH. Sex, age, BMI, hypertension, diabetes, smoking history, drinking history, preoperative antiplatelet/anticoagulant use, gross total resection (GTR), cystic tumor, postoperative INR, CNS infection, and mortality did not affect POH. And logistic regression analysis indicated that tumor diameter >30 mm [odds ratio (OR) 3.192, 95% confident interval (CI) 1.421–7.168, *P* = 0.005] and PTE (OR 4.042, 95% CI 1.830–8.926, *P* = 0.001) were independent predictive factors for POH.

**Table 2:** Univariate analysis of variables associated with reoperation and hemorrhage.

**Variables**	**Non-hemorrhage group (*n* = 415)**	**Hemorrhage group (*n* = 37)**	***P***	**Non-reoperation group (*n* = 438)**	**Reoperation group (*n* = 14)**	***P***
**Demographics**						
Sex (Male/Female)	185/230	20/17	0.303	197/241	8/6	0.421
Age, years (Median ± IQR)	53.0 ± 16.0	53.0 ± 15.5	0.518	53.0 ± 16.0	58.5 ± 11.0	0.109
BMI, kg/m^2^ (Median ± IQR)	23.0 ± 4.2	22.6 ± 4.7	0.796	23.0 ± 4.2	22.5 ± 5.5	0.319
**Past history**						
Hypertension	117 (28.2%)	11 (29.7%)	0.850	125 (28.5%)	3 (21.4%)	0.766
Diabetes	23 (5.5%)	2 (5.4%)	1.000	25 (5.7%)	0 (0%)	1.000
Smoking history	133 (32.0%)	10 (27.0%)	0.585	140 (32%)	3 (21.4%)	0.563
Drinking history	99 (23.9%)	11 (29.7%)	0.549	104 (23.7%)	6 (42.9%)	0.116
Preoperative antiplatelet or anticoagulant use	3 (0.7%)	0 (0%)	1.000	3 (0.7%)	0 (0%)	1.000
**Tumor characteristics**						
PTE	35 (8.4%)	13 (35.1%)	** <0.001**	42 (9.6%)	6 (42.9%)	**0.002**
Max tumor diameter > 30 mm	175 (42.2%)	28 (75.7%)	** <0.001**	192 (43.8%)	11 (78.6%)	**0.013**
Max tumor diameter, mm (Median ± IQR)	28.9 ± 14.3	39.6 ± 12.1	** <0.001**	29.0 ± 15.0	37.2 ± 10.8	**0.015**
GTR	281 (67.7%)	20 (54.1%)	0.103	294 (67.1%)	7 (50%)	0.248
Cystic tumor	190 (45.8%)	19 (51.4%)	0.606	200 (45.7%)	9 (64.3%)	0.185
**Postop. clinical characteristics**						
Severe hypertension	22 (5.3%)	15 (40.5%)	** <0.001**	30 (6.8%)	7 (50%)	** <0.001**
INR (Median ± IQR)	1.04 ± 0.10	1.06 ± 0.09	0.062	1.04 ± 0.10	1.07 ± 0.11	0.300
CNS infection	32 (7.7%)	3 (8.1%)	1.000	32 (7.3%)	3 (21.4%)	0.087
Death	1 (0.2%)	1 (2.7%)	0.157	1 (0.2%)	1 (6.7%)	0.061
Length of hospital stay, days (Median ± IQR)	16.0 ± 6.0	21.0 ± 9.5	** <0.001**	16.0 ± 6.0	27.5 ± 15.3	** <0.001**

*IQR, interquartile range; PTE, peritumoral edema; GTR, gross total resection; INR, international normalized ratio; CNS, central nervous system. The bold values represent P < 0.05*.

The univariate analysis also revealed that PTE (*P* = 0.002), max tumor diameter (*P* = 0.013), tumor diameter >30 mm (*P* = 0.013), severe postoperative hypertension (*P* < 0.001), and length of hospital stay (*P* < 0.001) were associated with reoperation for POH. Other factors had no statistical relation with reoperation as shown in Table 3, we found that PTE (OR 7.071, 95% CI 2.342–21.356, *P* = 0.001) was an independent predictive factor for reoperation.

**Table 3:** Logistic regression analysis identifying independent predictive factors of POH and reoperation.

**Variables**	**Hemorrhage**			**Reoperation**		
	**OR**	**95%CI**	***P***	**OR**	**95%CI**	***P***
PTE	4.042	1.830–8.926	**0.001**	7.071	2.342–21.356	**0.001**
Max tumor diameter > 30 mm	3.192	1.421–7.168	**0.005**	NA	NA	0.079

*OR, odds ratio; CI, confidence interval; PTE, peritumoral edema; NA, not applied. The bold values represent P < 0.05*.

### Correlation Between PTE and Other Tumor Characteristics

**Figure 3:**
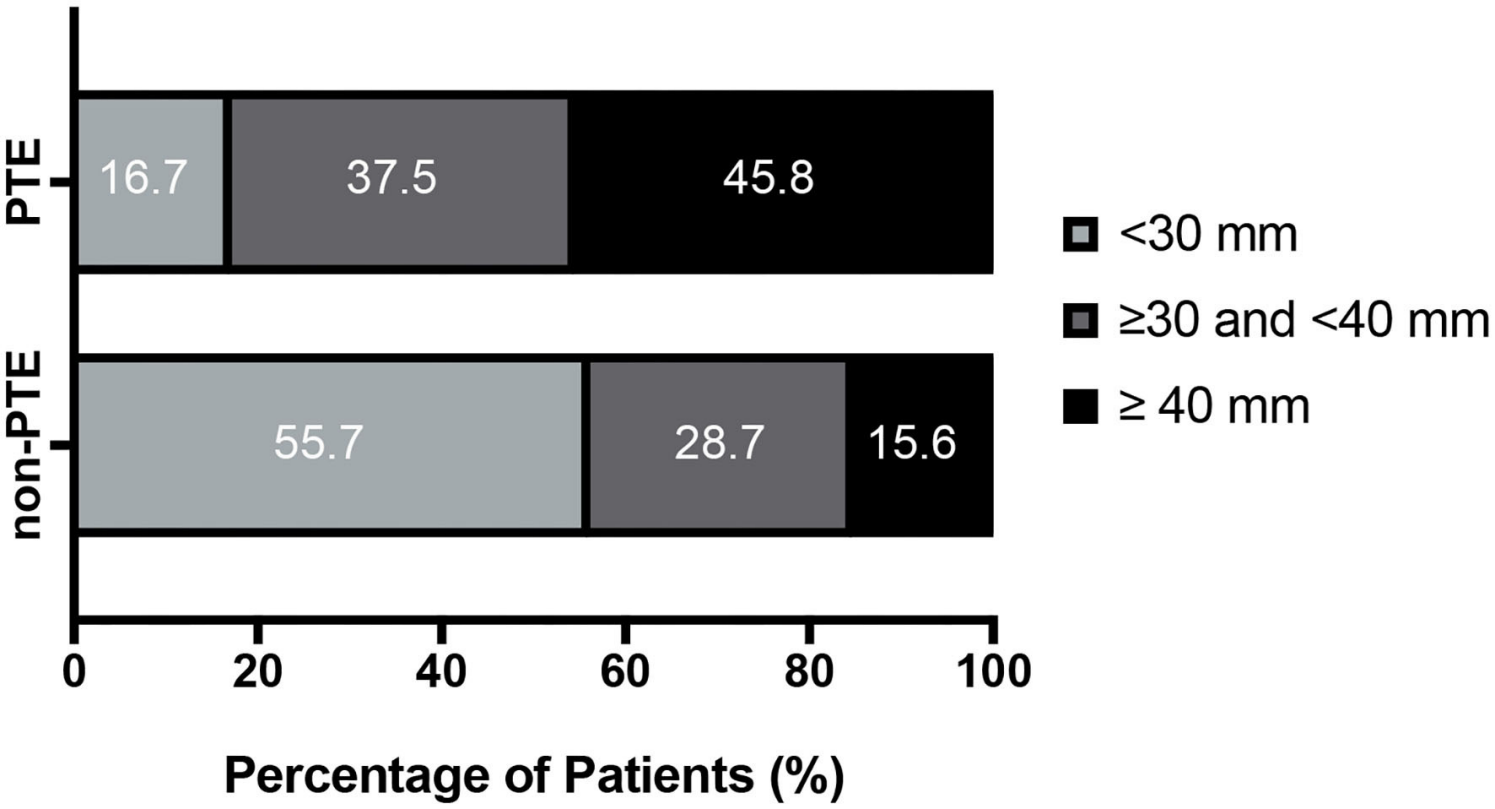
Tumor size and peritumoral edema (PTE).

Given the significant effects of PTE, 452 patients were divided into PTE group and non-PTE group based on the presence or absence of PTE. As shown in Table 4, median max tumor diameter was 38.9 ± 11.0 mm for PTE group and 28.0 ± 14.4 mm for non-PTE (*P* < 0.001). In addition, the patients were categorized according to max tumor diameter (<30 mm, 30–40 mm, and ≥40 mm, respectively). The results were shown in the Figure 3 and Table 4 (*P* < 0.001). PTE was also associated with incomplete tumor resection (*P* < 0.001). But, no statistically significant differences were observed regarding the ratio of cystic tumor between the two groups.

**Table 4:** Correlation between PTE and other tumor characteristics.

**Variables**	**PTE group (*n* = 48)**	**Non-PTE group (*n* = 404)**	***P***
Max tumor diameter, mm (Median ± IQR)	38.9 ± 11.0	28.0 ± 14.4	** <0.001**
GTR	20 (41.7%)	281 (69.6%)	** <0.001**
Cystic tumor	24 (50%)	185 (45.8%)	0.647
Max tumor diameter			** <0.001**
<30 mm	8 (16.7%)	225 (55.7%)	
≥30 and <40 mm	18 (37.5%)	116 (28.7%)	
≥40 mm	22 (45.8%)	63 (15.6%)	

*PTE, peritumoral edema; GTR, gross total resection. The bold values represent P < 0.05*.

## Discussion

This is the first study to identify the risk factors of POH and reoperation following the resection of vestibular schwannoma. We found that POH was not uncommon (8.2%), although most of hemorrhagic patients didn't require reoperation. The incident rate of POH seemed higher than those previously reported (1–3, 5, 11). We supposed that the majority of hemorrhagic patients were asymptomatic or paucisymptomatic, which might not be reported in previous studies. The reoperation rate (3.1%) was also slightly higher than those in previous studies which varied from 1.5 to 2.8% (2, 11). POH, as a severe complication, significantly affects patients' recovery and may even lead to death in some patients (3). We believe it is important for surgeons, especially for novice surgeons, to raise their attention to risk factors before surgery to reduce the incidence of POH and reoperation and further improve surgical quality.

PTE was an independent risk factor for POH and reoperation following VS resection. The incidence of PTE in VS varied from 5 to 38% (12, 13). In our study, the incidence of PTE (10.6%) was similar to the study performed by Samii and his colleagues. They found VS with PTE was more hypervascular and had a higher risk of postoperative intracranial hemorrhage. This finding was consistent with our study. Although it is still unclear why VS with PTE is prone to cause bleeding, the phenomenon that VS with PTE is more hypervascular suggests a certain molecular mechanism.

PTE was more widely recognized and frequently seen in meningioma. There was a study revealed that PTE was associated with tumor size in meningioma (14). A correlation between the incidence of PTE and tumor size was also found in VS (15). For meningioma, a possible explanation was that larger tumors could compress the normal brain tissues. In our study, although further analysis supported that PTE might be associated with tumor size, it was not observed in most VS with considerable size or significant mass effect. In addition, one study revealed that tumor volume and PTE volume were not relevant, which ought to show an association according to cerebral compressive theory (15). Another explanation was that vascular endothelial growth factor may serve an important role in the process of PTE in meningioma, including permeating into normal brain tissues, promoting PTE and feeding tumors via intracranial vessels (16, 17). Besides, PTE in meningioma was associated with loss of the arachnoid plane (18). As for PTE in VS, similar mechanisms may help to explain why PTE was associated with incomplete tumor resection (*P* < 0.001). Meanwhile, we suppose that this hypothesis can also contribute to explain the correlation between the incidence of PTE and tumor size, because only larger VS can contact with normal brain parenchyma.

Tumor size was the other independent risk factor for POH. Generally speaking, larger VS has a higher risk of postoperative hemorrhagic complications, since the draining veins in CPA may be engorged and fragile (4). In addition, subtotal resection is usually not only performed to prevent facial nerve damage but also to preserve other vital CPA structures when intraoperative bradycardia, hypotension or hypertension occurs (19, 20). However, subtotal or partial resection of VS are prone to hemorrhage, which may require reoperation. Previous studies also showed that the size of VS could significantly affect the incidence of POH, which was consistent with our findings (4, 21, 22).

In our study, the reoperation group had a higher rate of severe postoperative hypertension than the non-reoperation group (50 vs. 6.8%, *P* < 0.001). Basali et al. (23) reported that 62% of the POH patients after craniotomy had pre-hemorrhage hypertension in the early postoperative period. In our study, severe postoperative hypertension occurred prior to or simultaneously with deterioration of vital signs or worsening of neurological symptoms in five patients (5/7). But at present, it is still unclear whether severe postoperative hypertension is the cause or the consequence of POH, since the researches about postoperative hypertension and hemorrhage are not abundant. And the exact temporal relationship between the postoperative hypertension and hemorrhage can be determined by further studies. Besides, in the seven patients with severe postoperative hypertension in the reoperation group, only one of them (14.3%) had a history of hypertension. While in the non-reoperation group of 30 patients, a history of hypertension was found in 21 of them (70%). We suspect that the patients presenting with severe postoperative hypertension but without a history of hypertension are more prone to suffer POH, which should be paid more attention to. In a word, continuous blood pressure monitoring and precise blood pressure control may be helpful for preventing POH and reducing the risk of reoperation.

Our study still has several limitations. First, the most important limitation of our study is the sample size. We acknowledge the relative small sample size of reoperation group may lead to potentially false positive. Second, this study may be limited by its retrospective design. Although the radiological data was recorded by two independent neurosurgeons, its inherent bias was inevitable. Further prospective studies with larger samples may strengthen our findings.

In conclusion, PTE and tumor size played a critical role in the emergence and development of POH. And PTE was an independent predictive factor of reoperation for POH. Tumor size and extent of tumor resection may be associated with PTE. Close attention should be paid to high-risk patients especially for those who presented with severe postoperative hypertension.

## Data Availability Statement

The raw data supporting the conclusions of this article will be made available by the authors, without undue reservation.

## Ethics Statement

The studies involving human participants were reviewed and approved by the Human Ethics Review Committee of the Second Affiliated Hospital, Zhejiang University School of Medicine. Written informed consent for participation was not required for this study in accordance with the national legislation and the institutional requirements.

## Author Contributions

XG contributed to the study design, data collection and analysis, and drafting the manuscript. YZ contributed to the data analysis and drafting the manuscript. XW and KX collected the data. YH designed the study and critically revised the manuscript. All authors contributed to the article and approved the submitted version.

## Conflict of Interest

The authors declare that the research was conducted in the absence of any commercial or financial relationships that could be construed as a potential conflict of interest.
